# Molecular epidemiological characteristics of H9N2 subtype avian influenza virus in the external environment of western Zhejiang, China, 2014–2025

**DOI:** 10.3389/fpubh.2026.1839754

**Published:** 2026-08-12

**Authors:** Shiteng Huang, Bingdong Zhan, Min Wang, Lei Lyu, Jialing You, Ruijun Yang, Hui Yang

**Affiliations:** 1 Department of Microbiology Laboratory, Quzhou Center for Disease Control and Prevention, Quzhou, Zhejiang, China; 2 Department of Infectious Disease Control, Quzhou Center for Disease Control and Prevention, Quzhou, Zhejiang, China

**Keywords:** avian influenza virus, external environment, genetic evolution, H9N2 subtype, molecular characteristics

## Abstract

**Objective:**

To elucidate the epidemiological distribution patterns of avian influenza virus (AIV) in the external environment of western Zhejiang from 2014 to 2025, analyze the molecular epidemiological characteristics of the H9N2 subtype, and assess its public health risks.

**Methods:**

According to the Zhejiang Provincial Surveillance Program for Avian Influenza in Occupationally Exposed Populations and External Environments, real-time RT-PCR was used to detect AIV subtypes in environmental specimens. H9N2-positive samples with cycle threshold values <30 were inoculated into specific pathogen-free (SPF) embryonated chicken eggs for virus isolation, followed by whole-genome sequencing and bioinformatics analysis for phylogenetic and molecular characterization.

**Results:**

A total of 7,762 specimens were tested from 2014 to 2025, with an overall positivity rate of 34.64% (2,689/7,762) for AIV. Significant differences in positivity rates were observed in seasons, regions, sampling sites, and specimen types (all *p* < 0.001). AIV activity peaked in winter and spring, with the highest rates detected in live poultry markets and chopping board swabs. The H9 was the predominant subtype, with co-circulation of multiple subtypes. All 48 H9N2 subtype isolates belonged to the G57 genotype, with the hemagglutinin (HA) and neuraminidase (NA) genes falling into the Y280-like branch, while the internal genes exhibited a mosaic pattern combining G1-like and F/98-like lineages. Molecular characterization analysis revealed multiple mammalian adaptive mutations, involving alterations in receptor-binding sites (T163N, H191N, T197D, T198V, Q234L, Q235M), antigenic epitopes (D280G, N285S), and glycosylation sites (218NRTF, 313NCSK). NA stalk deletion (62–64 aa), along with multiple mutations in the hemadsorption site (E/K368N, D369S/G, D401G/V, N402D, W403L/R, Q432H). Additionally, multiple key amino acid substitutions were also identified in the internal proteins.

**Conclusion:**

The external environment in western Zhejiang exhibits a high prevalence of AIVs with pronounced spatiotemporal clustering. H9 was the dominant subtype and co-circulated with multiple subtypes, with live poultry markets and slaughterhouses identified as high-risk settings. The H9N2 subtype AIV has accumulated multiple mammalian adaptive mutations, and exhibits genetic linkages across eastern Chinese provinces. These findings collectively underscore the need for an integrated One Health surveillance and early-warning system to reduce the risk of human infections with avian influenza.

## 1 Introduction

Avian influenza is a zoonotic disease caused by avian influenza virus (AIV). Based on pathogenicity in poultry, AIV can be classified into highly pathogenic avian influenza virus (HPAIV) and low pathogenic avian influenza virus (LPAIV) (1). Currently, the AIV subtypes that possess the ability to directly infect humans are predominantly H5, H7, and H9. Among these, certain strains within the H5 and H7 subtypes are classified as HPAIV, capable of causing large-scale outbreaks and resulting in high mortality in poultry and even humans. In contrast, the H9N2 subtype is classified as LPAIV; nevertheless, owing to its high transmissibility, H9N2 has become globally widespread and is currently the most widely distributed AIV subtype with the highest detection rate (2), thereby posing a serious threat to global public health security. To date, China has reported the highest number of human H9N2 infections worldwide, with an increasing trend in case numbers (3, 4).

H9N2 subtype can not only directly infect humans but also serve as a “gene donor” to undergo genetic reassortment with other AIV subtypes, facilitating the emergence of novel subtypes and posing a potential threat of causing an avian influenza pandemic (5). Epidemiological studies have revealed that the internal genes of HPAIV such as H7N9, H5N6, and H10N8 originated from the H9N2 subtype (6, 7). Therefore, in-depth researches into the epidemiological patterns and genetic evolutionary characteristics of the H9N2 subtype AIV are of great significance for the prevention and control of avian influenza, as well as for safeguarding public health security.

The external environment serves as a critical reservoir for the transmission, persistence, and evolution of AIV, constituting a key node in the poultry-environment-human cycle and representing a significant source of human infection (8). Therefore, Surveillance of AIV in the external environment constitutes a core strategy for elucidating the ecological distribution of the virus and for early warning of cross-species transmission risks. Western Zhejiang, as an important ecological barrier in the Yangtze River Delta and a region with intensive poultry breeding, represents a priority region for AIV surveillance.

Previous studies on AIV in this region have been constrained by several limitations: (1) most investigations covered only short periods (typically 1–3 years) or discrete time points, precluding robust temporal trend analysis; (2) analyses were predominantly univariate, without multivariable adjustment for confounding factors such as season, location, sampling site, and specimen type; and (3) molecular characterization was often limited to partial gene segments rather than complete genomes. Accordingly, this study provides three unique contributions. First, it presents the longest continuous environmental surveillance dataset for AIV in western Zhejiang to date (2014–2025, 12 years), enabling robust spatiotemporal trend analysis via Cochran–Armitage trend tests; Second, it integrates multivariable logistic regression to simultaneously delineate the independent effects of season, geography, sampling site type, and specimen type on AIV positivity—a multidimensional analytical framework not previously applied to this region; Third, it systematically characterizes the full-genome molecular evolution of local H9N2 isolates, linking accumulating mammalian-adaptive mutations to cross-provincial genetic networks spanning Zhejiang, Fujian, Jiangxi, and Anhui. These findings provide actionable molecular markers for One Health early-warning systems and a scientific basis for regional avian influenza prevention and control.

## 2 Materials and methods

### 2.1 Specimen sources

According to the requirements of the Zhejiang Provincial Surveillance Program for Avian Influenza in Occupationally Exposed Populations and External Environments (2014–2025). Monitoring sites were selected if they met at least one of the following criteria: (1) previous human or animal avian influenza outbreaks in the area or adjacent regions; (2) dense water networks (lakes, rivers) and intensive waterfowl production in surrounding areas; (3) location within a migratory bird habitat or flyway; (4) high prevalence of free-range poultry holdings. The monitoring sites in western Zhejiang included Kecheng District, Qujiang District, Jiangshan City, Changshan County, Kaihua County, and Longyou County. Within each site, specimens were collected from the following environmental settings: urban and rural live poultry markets, large-scale poultry farms (households), areas with clustered backyard poultry households, as well as poultry slaughterhouses and processing plants.

Surveillance frequency and sample size: In the high-incidence season (October–March), environmental sampling is performed once every 2 weeks, with no fewer than 10 specimens of various types collected per sampling event. In the low-incidence season (April–September), sampling is performed once a month, with no fewer than 15 specimens of various types collected per sampling event. At each monitoring site, specimens should be collected from at least 2–3 sampling locations. The sample types included poultry feces, poultry drinking water, swabs from the surfaces of chopping boards used for slaughtering or displaying poultry, cage surface swabs, and poultry cleaning wastewater.

### 2.2 RT-PCR and virus isolation

Viral RNA was extracted from 200 μL of each sample using the CqEx-RNA/DNA Virus (CDC) kit (Tianlong Technology Co., Ltd., Xi’an, China). Influenza A virus nucleic acid was detected using a real-time RT-PCR kit for influenza virus (Jiangsu Bioperfectus Technologies Co., Ltd.) (Ct values less than 38 were considered positive, while those with Ct values between 38 and 40 were subjected to repeat experiments due to the requirement for confirmation. Samples with Ct values greater than 40 were regarded as negative). Positive specimens were further subtyped for H5, H7, H9, N2, and N9 using corresponding real-time RT-PCR kits. H9N2-positive samples with Ct values <30 were selected for virus isolation. These specimens were diluted 1:1 with a penicillin–streptomycin PBS solution, filtered (0.22 μm) and then 200 μL of the filtrate were inoculated into 9-11-day-old specific pathogen-free (SPF) embryonated chicken eggs. Allantoic fluid was collected 48–72 h post inoculation and then identified by real-time RT-PCR and hemagglutination assays. Isolates confirmed as H9N2 subtype with a hemagglutination titer of ≥8 were selected for subsequent analyses. A total of 48 H9N2 isolates were successfully obtained, spanning all 12 surveillance years (2014–2025), thereby providing a temporally representative panel for genomic analysis.

### 2.3 Whole-genome sequencing and sequence analysis

According to *the National Influenza Surveillance Technical Guide (2017 Edition)*, whole-genome specific amplification was performed using the ULSEN^®^ Ultra-Sensitive Influenza A Virus Whole-Genome Capture Kit (Beijing MicroFuture Technology Co., Ltd.). A whole-genome sequencing library was constructed with the NGSmaster Metagenomics One-Step Library Preparation System (Hangzhou Matridx Biotechnology Co., Ltd.), and whole-genome sequencing of the H9N2 subtype was conducted on the Illumina MiSeq sequencing platform (Illumina, Inc., USA), with a targeted average depth of coverage ≥100 × per genomic segment. Quality control of the NGS reads was assessed using CLC Genomic Workbench v10 (QIAGEN，Germany). Low quality reads less than 50 nucleotides in length were removed and a minimum base call quality Phred score of 20 was set.

Sequence assembly was performed using the Hangzhou Baiyi Pathogen Analysis Software (Baiyi Technology Co., Ltd.). The assembly parameters were set as follows: minimum contig length 200 bp, minimum coverage ≥10×, and identity threshold ≥95% for reference-based mapping. Reference sequences were obtained from the Global Initiative on Sharing All Influenza Data (GISAID) (9), including classic strains (A/chicken/Beijing/1/1994, A/quail/HongKong/G1/1997, A/duck/HongKong/Y439/1997, and A/duck/Hongkong/Y280/1997), vaccine strains (A/chicken/Shandong/6/1996, A/Chicken/Guangdong/SS/1994, A/chicken/Shanghai/F/1998, A/Hong Kong/308/2014, and A/Anhui-Lujiang/39/2018), and human-derived strains (A/Fujian-siming/19/2021 and A/Anhui-Langya/1144/2022). The hemagglutinin (HA) and neuraminidase (NA) gene sequences of the isolates were compared via BLAST against the reference sequences from the GISAID database. In this study, an E-value threshold of ≤1 × 10^−5^ was applied to define significant homology, consistent with widely accepted standards for BLAST-based homology inference. Molecular characterization of the eight gene segments was conducted using MegAlign module in DNAStar 7.0.1 (Lasergene, USA) (10). Multiple sequence alignments were generated with MUSCLE (default parameters) and manually edited if necessary. Phylogenetic trees were constructed using MEGA 7.0.26 with the neighbor-joining (NJ) method and 1,000 bootstrap replicates (11). Genotype nomenclature followed the classification system established by Pu et al. (2015) (12). Potential N-linked glycosylation sites in the amino acid sequences of HA and NA proteins were predicted and analyzed using the online tool NetNGlyc 1.0 Server (NetNGlyc 1.0 - DTU Health Tech - Bioinformatic Services, Denmark) (13). The default prediction threshold of 0.50 was applied to define a positive N-glycosylation site, meaning that only asparagine residues with a potential score ≥0.50 were considered putative glycosylation sites.

### 2.4 Statistical analysis

A database was established and organized using Excel 2016, and statistical analysis was performed using R software (version 4.2.1, R Foundation for Statistical Computing, Vienna, Austria). The chi-square test was employed to examine differences in AIV positivity rates in different years, seasons, regions, sampling sites, and specimen types. The Cochran-Armitage trend test and logistic regression were applied to evaluate the statistical significance of linear trends in AIV positivity rates across the 12-year surveillance period (2014–2025). Multivariable logistic regression was used to identify independent predictors of AIV positivity. All four categorical variables (quarter, district, sampling site, and specimen type) were entered into a single model simultaneously. The reference category for each variable was selected based on its role as the baseline in field surveillance: first quarter, Kecheng District, scale farms, and cage surfaces. Adjusted odds ratios (*aOR*) and 95% confidence intervals (95% *CI*) were reported. *p*-value < 0.05 was considered statistically significant.

## 3 Results

### 3.1 Surveillance summary

**Figure 1:**
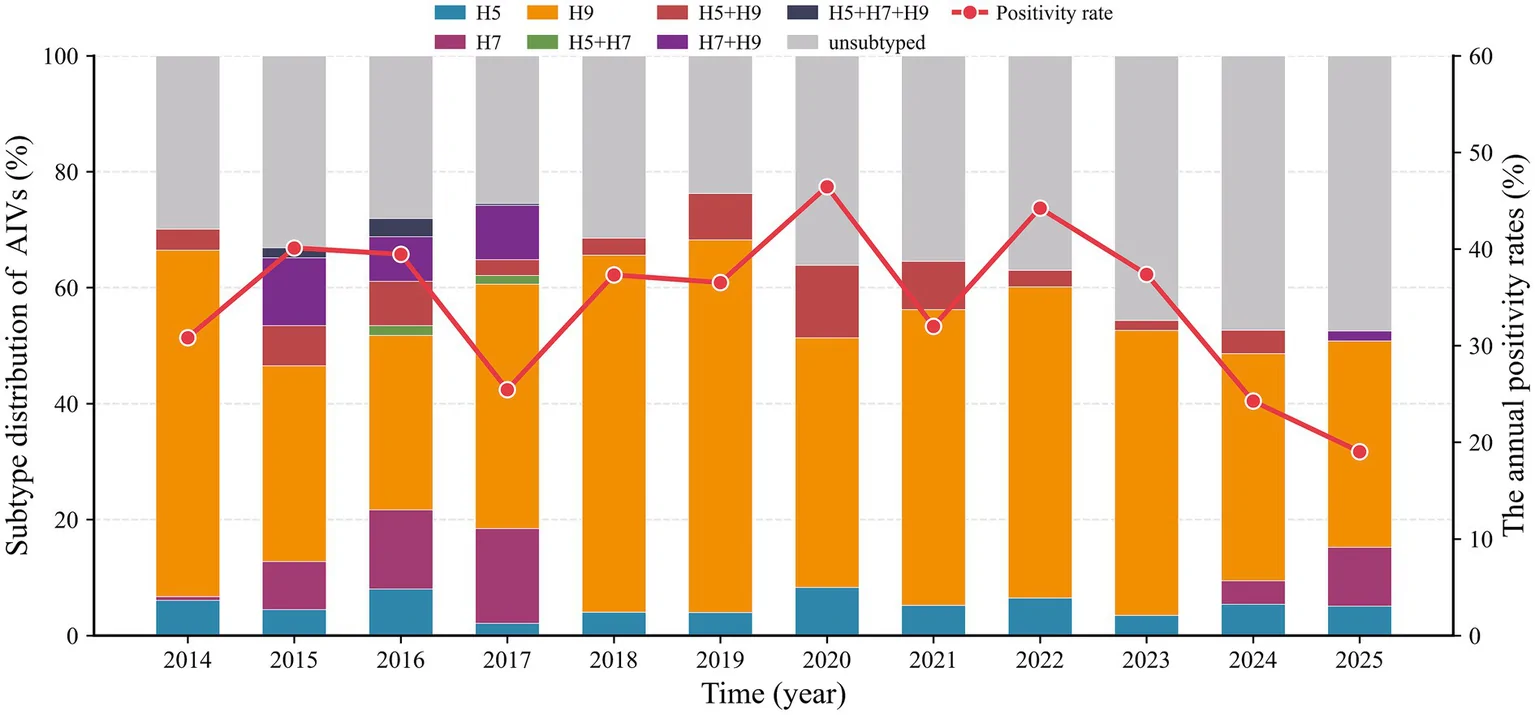
Detection rate and subtype distribution of avian influenza viruses in external environment in western Zhejiang, 2014–2025.

Between 2014 and 2025, a total of 7,762 external environmental specimens were collected in western Zhejiang, of which 2,689 tested positivity for AIV, yielding an overall positivity rate of 34.64%. The annual positivity rates were 30.83% (164/532), 40.11% (290/723), 39.45% (535/1356), 25.44% (330/1297), 37.32% (471/1262), 36.53% (274/750), 46.45% (144/310), 32.00% (96/300), 44.23% (138/312), 37.38% (114/305), 24.26% (74/305), and 19.03% (59/310), respectively, showing a statistically significant difference (*χ^2^* = 162.09, *p* < 0.001). Among the 2,689 AIV-positive specimens, the distribution proportions of different subtypes were as follows: H5 subtype accounted for 5.21% (140/2,689), H7 subtype for 5.99% (161/2,689), H9 subtype for 46.60% (1,253/2,689), H5 + H7 mixed infections for 0.52% (14/2,689), H5 + H9 mixed infections for 5.47% (147/2,689), H7 + H9 mixed infections for 3.98% (107/2,689), and H5 + H7 + H9 mixed infections for 0.86% (23/2,689), while unsubtyped AIV accounted for 31.39% (844/2,689). The differences in detection rates among subtypes and co-infections were statistically significant (*χ^2^* = 4,956.69, *p* < 0.001), with the H9 subtype was identified as the predominant prevalent subtype, as shown in Figure 1. To assess the overall temporal trend in AIV positivity, the Cochran-Armitage trend test was applied at the individual sample level (*n* = 7,762). A statistically significant downward trend was identified across the entire study period (2014–2025; *Z* = −2.90, *p* = 0.004). To further evaluate whether the trend differed between sub-periods, a logistic regression model with a period × year interaction term was fitted. During 2014–2020, no significant trend was observed (*OR* = 1.026 per year, 95% *CI*: 0.993–1.060, *p* = 0.120), whereas a marked and significant decline was identified during 2021–2025 (*OR* = 0.803 per year, 95% *CI*: 0.743–0.869, *p* < 0.001). The interaction term was highly significant (*OR* = 0.783, 95% *CI*: 0.720–0.852, *p* < 0.001), confirming that the rate of decline was significantly greater in the latter period, suggesting an accelerating reduction in environmental AIV contamination in recent years.

**Figure 2:**
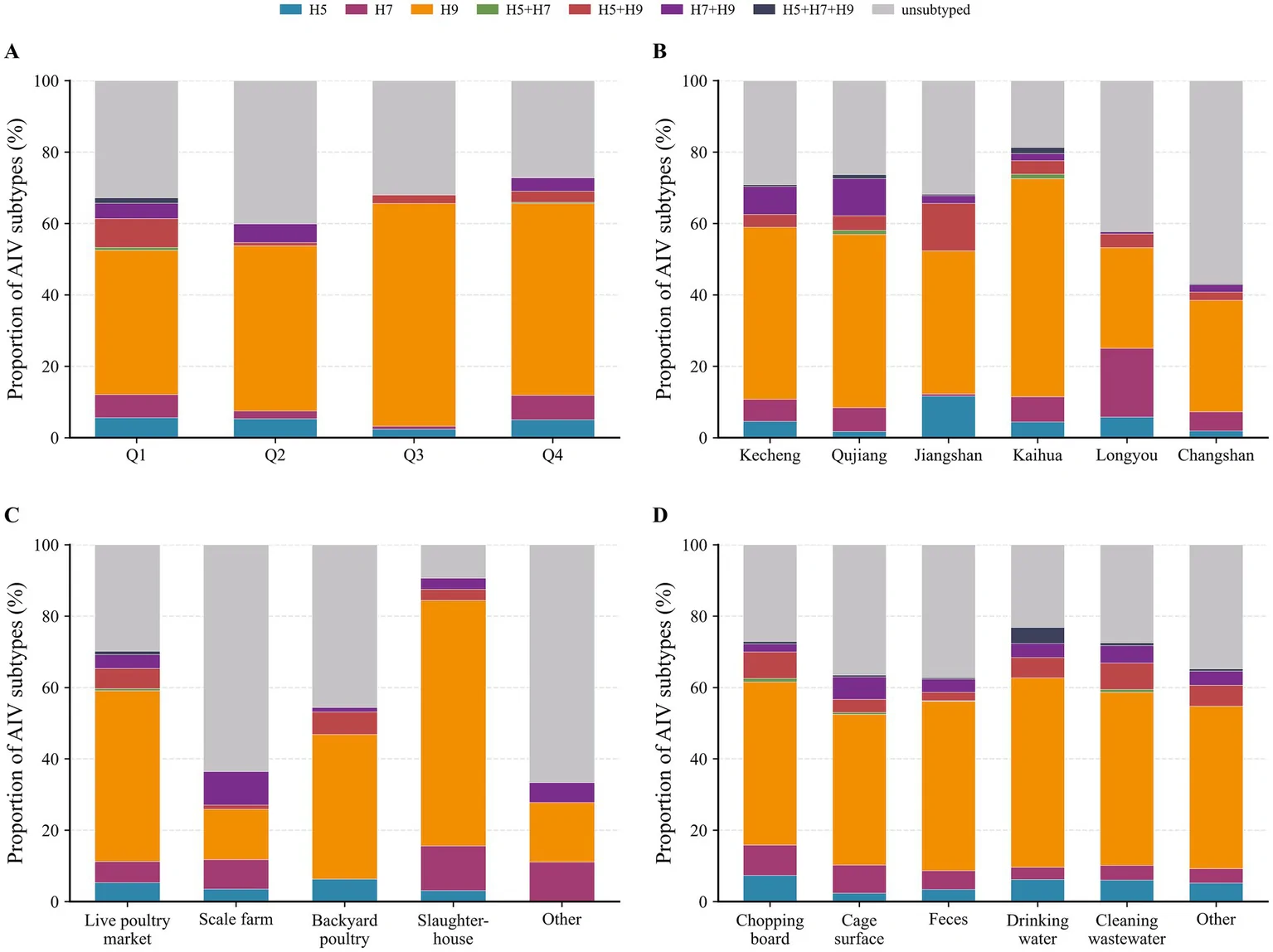
Distribution of avian influenza virus subtypes by quarter **(A)**, region **(B)**, sampling site **(C)**, and specimen type **(D)**.

Table 1 presents statistically significant differences in AIV positivity rates in different seasons, regions, sampling sites, and specimen types (all *p* < 0.001). Notably, the H9 subtype and unsubtyped AIV were the predominant types in all categories (Figure 2). Specifically, the highest positivity rate was observed in the first quarter (39.04%), followed by the fourth quarter (36.69%). In terms of regional distribution, Kaihua County recorded the highest positivity rate (60.92%), followed by Jiangshan City (41.30%). Regarding sampling sites, urban and rural live poultry markets exhibited the highest positivity rate (49.28%), followed by poultry slaughterhouses and processing plants (42.67%). For specimen types, swab samples collected from the surface of chopping boards used for slaughtering or displaying poultry showed the highest positivity rate (60.88%), followed by wastewater samples from poultry cleaning (46.30%). Multivariable logistic regression identified several independent predictors of AIV positivity (reference categories: first quarter, Kecheng District, scale farms, cage surfaces). Positivity was significantly lower in second quarter (*aOR* = 0.66, 95% *CI*: 0.55–0.80) and third quarter (*aOR* = 0.33, 95% *CI*: 0.26–0.41) compared with first quarter. Geographically, Kaihua County (*aOR* = 3.89, 95% *CI*: 3.22–4.69) and Jiangshan City (*aOR* = 1.89, 95% *CI*: 1.57–2.26) had the highest risk. At the sampling-site level, live poultry markets (*aOR* = 6.44, 95% *CI*: 5.18–8.02) and slaughterhouses (*aOR* = 4.59, 95% *CI*: 2.64–7.98) were the strongest independent predictors of positivity. For specimen type, cutting board surfaces (*aOR* = 3.34, 95% *CI*: 2.72–4.10) and cleaning wastewater (*aOR* = 2.25, 95% *CI*: 1.83–2.76) yielded higher positivity than cage surfaces, while feces and drinking water were associated with lower rates.

**Table 1:** Surveillance results of avian influenza virus in external environmental specimens in western Zhejiang, 2014–2025.

Variables	No. tested	No. (%) positive	Univariate analysis		Multivariate analysis	
			*χ^2^*-value	*p*-value	aOR(95%CI)	*p*-value
Quarter			177.12	<0.001		
First	3,586	1,400 (39.04)			1	
Second	840	227 (27.02)			0.66(0.55–0.80)	<0.001
Third	782	125 (15.98)			0.33 (0.26–0.41)	<0.001
Fourth	2,554	937 (36.69)			0.96 (0.85–1.09)	1.000
Region			714.33	<0.001		
Kecheng District	1,404	419 (29.84)			1	
Qujiang District	1,191	346 (29.05)			0.88 (0.73–1.07)	1.000
Jiangshan City	1,310	541 (41.30)			1.89 (1.57–2.26)	<0.001
Kaihua County	1,310	798 (60.92)			3.89 (3.22–4.69)	<0.001
Longyou County	1,215	156 (12.84)			0.34 (0.28–0.43)	<0.001
Changshan County	1,332	429 (32.21)			1.16 (0.96–1.39)	1.000
Sampling site			1400.02	<0.001		
Large-scale poultry farms	769	85 (11.05)			1	
urban and rural live poultry markets	5,022	2,475 (49.28)			6.44 (5.18–8.02)	<0.001
Areas with clustered backyard poultry farm	1746	79 (4.52)			0.26 (0.19–0.34)	<0.001
Poultry slaughterhouses and processing plants	75	32 (42.67)			4.59 (2.64–7.98)	<0.001
Other	150	18 (12.00)			1.02 (0.62–1.67)	1.000
Specimen type			649.39	<0.001		
Cage surface swabs	1,106	381 (34.45)			1	
Swabs from chopping board surfaces	1,158	705 (60.88)			3.34 (2.72–4.10)	<0.001
Poultry feces	2,684	588 (21.91)			0.62 (0.52–0.74)	<0.001
Poultry drinking water	742	177 (23.85)			0.59 (0.46–0.75)	<0.001
Poultry cleaning wastewater	1,108	513 (46.30)			2.25 (1.83–2.76)	<0.001
Other	964	325 (33.71)			1.04 (0.84–1.28)	1.000
Total	7,762	2,689 (34.64)				

*p*-values were adjusted using the Bonferroni correction for multiple comparisons. aOR = adjusted odds ratio; CI = confidence interval.

### 3.2 Phylogenetic analysis of H9N2 subtype isolates

In this study, a total of 48 H9N2 subtype AIV isolates were obtained from H9N2-positive samples with Ct values <30, spanning all 12 surveillance years (2014–2025) and covering the major high-risk districts (Kaihua County, Jiangshan City, Kecheng District, and others). All eight gene segments were successfully sequenced by whole-genome sequencing. The nucleotide and amino acid homologies of the HA segments among the isolates ranged from 90.94 to 100% and from 91.35 to 100%, respectively, while those of the NA segments ranged from 92.13 to 100% and from 92.41 to 100%, respectively. Compared with the human-derived strains A/Fujian-siming/19/2021 and A/Anhui-Langya/1144/2022, the HA segments exhibited nucleotide and amino acid homologies ranging from 89.57 to 98.73% and from 90.25 to 98.94%, respectively. BLAST analysis against the GISAID database revealed that the HA and NA gene sequences of the 48 H9N2 isolates shared the highest identities with reference sequences in the database, ranging from 98.14 to 99.78% and from 98.59 to 99.81%, respectively. The corresponding BLAST E-values for these top hits were all ≤1 × 10^−50^ for both HA and NA sequences, indicating extremely high statistical significance of the alignments. These reference sequences with the highest identities were derived from avian or human isolates originating from Zhejiang, Fujian, Jiangxi, Anhui, Shanghai, Henan, and Guangdong provinces in China, as shown in Table 2.

**Table 2:** BLAST analysis of HA and NA sequences of H9N2 subtype isolates from western Zhejiang against the GISAID database, 2014–2025.

Isolate	Strain with highest HA gene identity to the isolate	Identity (%)	Strain with highest NA gene identity to the isolate	Identity (%)
A/Environment/Zhejiang/C14190087/2014	A/chicken/Zhejiang/C19/2013	99.23	A/chicken/Zhejiang/925018/2014	99.51
A/Environment/Zhejiang/C14190148/2014	A/chicken/Zhejiang/925117/2014	99.31	A/chicken/Zhejiang/77001/2014	99.28
A/Environment/Zhejiang/C14190404/2014	A/chicken/China/D345/2013	99.22	A/chicken/Wenzhou/YHQL04/2014	99.59
A/Environment/Zhejiang/C14190432/2014	A/Environment/Zhenjiang/zj33/2014	99.37	A/chicken/Zhejiang/3C22/2013	98.93
A/Environment/Zhejiang/C15190037/2015	A/chicken/Fujian/FZHCAK-O/2015	99.09	A/chicken/China/F1370/2015	99.13
A/Environment/Zhejiang/C15190241/2015	A/Environment/Zhenjiang/zj33/2014	99.25	A/chicken/Jiangxi/19463/2014	99.21
A/Environment/Zhejiang/C15190367/2015	A/chicken/China/E2901/2014	99.78	A/chicken/China/E2620/2014	99.42
A/Environment/Zhejiang/C15190450/2015	A/Environment/Zhenjiang/zj33/2014	99.05	A/chicken/Jiangxi/19463/2014	99.17
A/Environment/Zhejiang/C15190589/2015	A/chicken/China/F282/2015	99.62	A/chicken/China/F1370/2015	99.35
A/Environment/Zhejiang/C16190068/2016	A/Chicken/Shanghai/F2170/2015	99.46	A/chicken/China/F344/2015	99.28
A/Environment/Zhejiang/C16190408/2016	A/chicken/Shanghai/S2022/2016	99.72	A/chicken/Zhejiang/12174/2016	99.34
A/Environment/Zhejiang/C16190674/2016	A/chicken/Zhejiang/52145/2016	99.23	A/chicken/Zhejiang/12174/2016	99.29
A/Environment/Zhejiang/C16190858/2016	A/chicken/Shanghai/S2022/2016	99.67	A/chicken/Zhejiang/1130150/2016	99.47
A/Environment/Zhejiang/C16190927/2016	A/chicken/Zhejiang/52145/2016	99.18	A/chicken/Zhejiang/12174/2016	99.21
A/Environment/Zhejiang/C17190059/2017	A/chicken/China/SCDC-SZ/2017	99.06	A/chicken/Jiangxi/NCNP3Q2-OC/2017	99.07
A/Environment/Zhejiang/C17190190/2017	A/Environment/Jiangxi/50756/2016	98.97	A/chicken/China/G591/2016	98.99
A/Environment/Zhejiang/C17190273/2017	A/chicken/China/SCDC-SZ/2017	99.11	A/chicken/Jiangxi/NCNP3Q2-OC/2017	99.09
A/Environment/Zhejiang/C17190508/2017	A/Environment/Jiangxi/50756/2016	99.03	A/chicken/Jiangxi/NCNP3Q2-OC/2017	99.05
A/Environment/Zhejiang/C17190736/2017	A/Chicken/Jiangxi/H1301/2017	98.94	A/chicken/China/G591/2016	99.11
A/Environment/Zhejiang/C17190842/2017	A/Chicken/Jiangxi/H1301/2017	98.96	A/chicken/China/G591/2016	99.15
A/Environment/Zhejiang/C18190055/2018	A/chicken/Zhejiang/HZBX017-O/2018	99.03	A/chicken/Zhejiang/HZBX007-C/2018	99.32
A/Environment/Zhejiang/C18190291/2018	A/chicken/Fujian/I33/2017	98.99	A/chicken/Zhejiang/HZBX003-C/2018	99.17
A/Environment/Zhejiang/C18190538/2018	A/chicken/China/A42/2018	99.12	A/chicken/Zhejiang/HZBX003-O/2018	99.81
A/Environment/Zhejiang/C18190636/2018	A/chicken/China/A42/2018	99.18	A/chicken/Zhejiang/HZBX007-C/2018	99.28
A/Environment/Zhejiang/C18190782/2018	A/chicken/Zhejiang/HZBX004-O/2018	99.07	A/chicken/China/1642.6/2018	99.09
A/Environment/Zhejiang/C18191163/2018	A/chicken/Fujian/F2134/2017	98.83	A/chicken/Guangdong/113/2019	98.96
A/Environment/Zhejiang/C19190029/2019	A/chicken/China/C7/2018	98.91	A/chicken/Zhejiang/X9-O/2019	99.21
A/Environment/Zhejiang/C19190037/2019	A/Chicken/Guangdong/GZ12363/2019	98.93	A/chicken/Fujian/FZHX0080-C/2018	99.07
A/Environment/Zhejiang/C19190089/2019	A/chicken/China/C7/2018	98.84	A/chicken/Zhejiang/X9-O/2019	99.15
A/Environment/Zhejiang/C19190113/2019	A/chicken/Zhejiang/X8-O/2019	99.05	A/chicken/Fujian/FZHX0080-C/2018	99.13
A/Environment/Zhejiang/C19190216/2019	A/chicken/Zhejiang/X8-O/2019	98.97	A/chicken/Zhejiang/X6-O/2019	99.04
A/Environment/Zhejiang/C20190011/2020	A/duck/Zhejiang/HZBX31-C/2020	99.75	A/chicken/Anhui/YHZGS007-O/2018	98.96
A/Environment/Zhejiang/C20190338/2020	A/chicken/Henan/WJX16-O/2021	98.97	A/chicken/China/2088/2020	98.81
A/Environment/Zhejiang/C20190773/2020	A/chicken/Henan/WJX16-O/2021	98.83	A/chicken/China/2088/2020	98.74
A/Environment/Zhejiang/C21190026/2021	A/environment/Xiamen/02/2021	98.75	A/chicken/Anhui/ZGS3-O/2021	99.02
A/Environment/Zhejiang/C21190455/2021	A/Fujian-siming/19/2021	98.73	A/chicken/Anhui/ZGS3-O/2021	98.95
A/Environment/Zhejiang/C21190752/2021	A/environment/Xiamen/02/2021	98.81	A/chicken/Zhejiang/ZJBX-O25/2021	99.08
A/Environment/Zhejiang/C21190802/2021	A/chicken/Fujian/FZHX68-O/2020	98.91	A/chicken/Anhui/ZGS3-O/2021	98.91
A/Environment/Zhejiang/C22190008/2022	A/chicken/Jiangxi/NCJD-O10/2021	98.84	A/chicken/Jiangxi/NCJD36-C/2021	99.13
A/Environment/Zhejiang/C22190462/2022	A/chicken/Anhui/ZGS7-O/2021	98.71	A/chicken/Anhui/ZGS3-O/2021	98.82
A/Environment/Zhejiang/C22190759/2022	A/duck/Jiangxi/NCJD-C7/2022	99.06	A/chicken/Jiangxi/NCJD36-C/2021	98.88
A/Environment/Zhejiang/C23190021/2023	A/duck/Jiangxi/M1563/2023	98.57	A/chicken/Jiangxi/M0679/2023	98.79
A/Environment/Zhejiang/C23190347/2023	A/chicken/China/434c6/2021	98.23	A/Env/Fujian/C22036088/2022	98.61
A/Environment/Zhejiang/C23190496/2023	A/duck/Jiangxi/M1563/2023	98.45	A/chicken/Jiangxi/M1719/2023	98.74
A/Environment/Zhejiang/C24190306/2024	A/environment/Xiamen/01/2021	98.31	A/Fujian-siming/1348/2020	98.59
A/Environment/Zhejiang/C24190463/2024	A/environment/Xiamen/01/2021	98.27	A/Fujian-siming/1348/2020	98.63
A/Environment/Zhejiang/C25190308/2025	A/chicken/Zhejiang/ZJBX-O17/2021	98.33	A/chicken/Zhejiang/ZJBX-O25/2021	98.71
A/Environment/Zhejiang/C25190504/2025	A/environment/Xiamen/01/2021	98.14	A/chicken/Fuzhou/C1/2021	98.62

**Figure 3:**
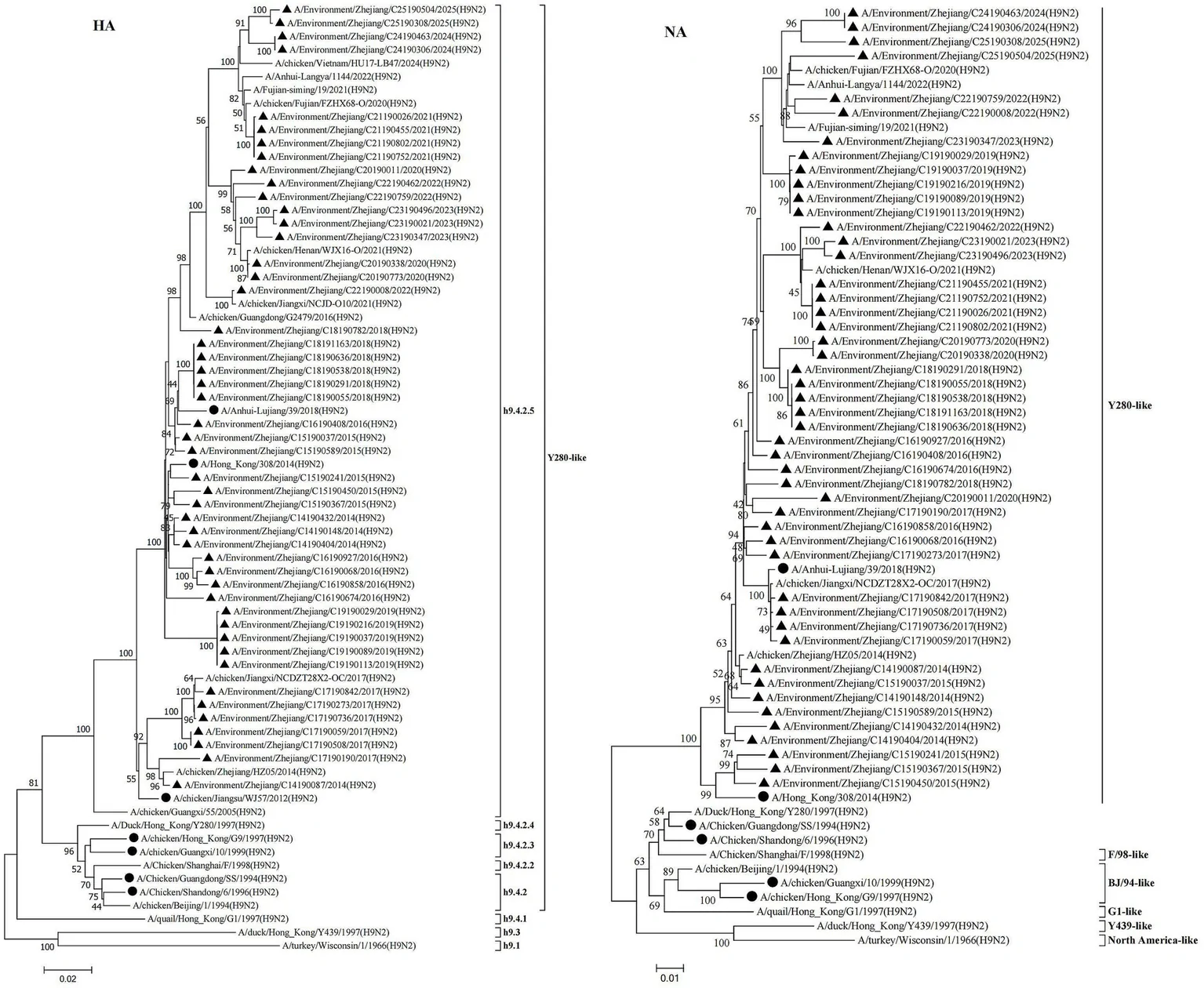
Phylogenetic tree of HA and NA genes of H9N2 subtype avian influenza virus isolates from external environments in western Zhejiang, 2014–2025. •Vaccine strains, ▴isolated strain in this study.

**Figure 4:**
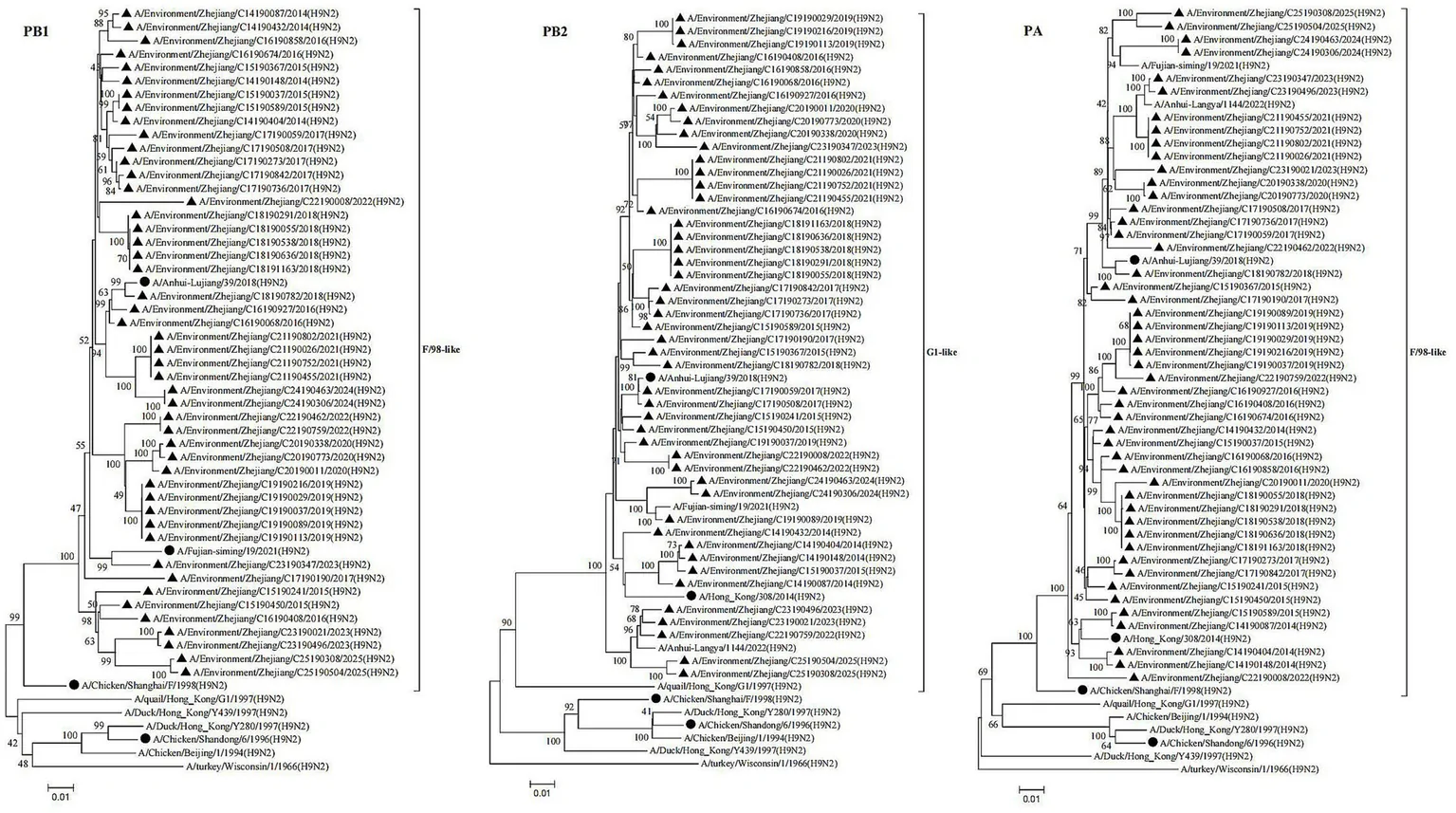
Phylogenetic tree of PB1, PB2, and PA genes of H9N2 subtype avian influenza virus isolates from external environments in Western Zhejiang, 2014–2025. •Vaccine strains, ▴isolated strain in this study.

**Figure 5:**
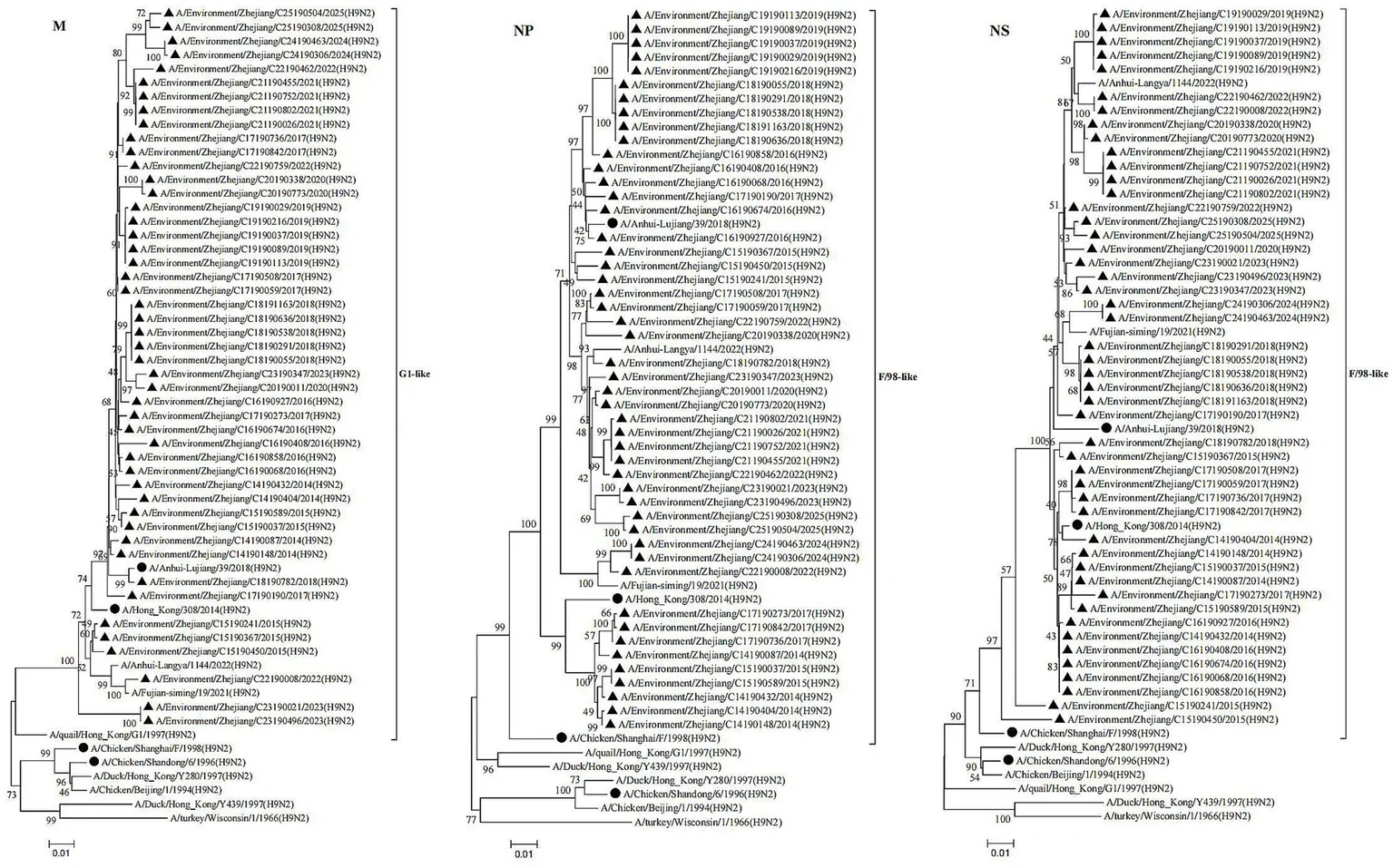
Phylogenetic tree of M, NP, and NS genes of H9N2 subtype avian influenza virus isolates from external environments in western Zhejiang, 2014–2025. •Vaccine strains, ▴isolated strain in this study.

Phylogenetic analysis revealed that all 48 H9N2 subtype isolates in this study were belonged to the G57 genotype according to the established classification criteria (12). The HA and NA genes clustered within the Eurasian lineage Y280-like branch, and were genetically close to the vaccine strains A/Hong Kong/308/2014 and A/Anhui-Lujiang/39/2018, as well as the human-derived strains A/Anhui-Langya/1144/2022 and A/Fujian-siming/19/2021, all clustering within the h9.4.2.5 sublineage, as shown in Figure 3. The polymerase basic protein 2 (PB2) and matrix protein (M) genes belonged to the G1-like branch, represented by A/quail/Hong Kong/G1/1997(H9N2). The polymerase basic protein 1 (PB1), polymerase acidic protein (PA), nucleoprotein (NP), and nonstructural protein (NS) genes belonged to the F/98-like branch, represented by A/chicken/Shanghai/F/1998(H9N2). This genetic constellation was consistent with the reassortment pattern of the dominant G57 genotype of H9N2 subtype AIV circulating in China since 2013, as shown in Figures 4, 5.

### 3.3 Key amino acid site analysis of H9N2 subtype isolates

In this study, the HA protein cleavage sites of the 48 H9N2 subtype isolates were identified as PSRSSR↓GLF (46 isolates), PSKSNR↓GLF (1 isolate), and PSRSNR↓GLF (1 isolate). None of the isolates contained multiple consecutive basic amino acids at the cleavage site, which is consistent with the characteristics of LPAIV. Alignment and analysis of key amino acid sites with classical strains, vaccine strains, and human-derived H9N2 strains revealed that all isolates carried mutations at receptor-binding sites in the HA protein, including H191N, Q234L, Q235M, and R381K (H9 numbering). Additionally, 13 isolates harbored the T163N mutation, 31 isolates carried the T197D mutation, and 20 isolates exhibited the T198V/A mutation. Antigenic epitope mutations D280G and N285S were also detected. All isolates displayed a 62–64 amino acid deletion in the stalk region of the NA protein. Mutations at the hemadsorption site included E/K368N, D369S/G, D401G/V, N402D, W403L/R, and Q432H. Among common neuraminidase inhibitor resistance-associated sites, only the N70S mutation was identified, which may reduce susceptibility to zanamivir (14), as shown in Table 3.

**Table 3:** Analysis of key amino acid sites of H9N2 subtype avian influenza virus isolates from external environments in western Zhejiang, 2014–2025.

Protein	Mutation site	48 isolates	A/chicken/Beijing/1/94	A/quail/HongKong/G1/97	A/duck/Hongkong/Y280/97	A/chicken/Shanghai/F/98	A/chicken/Shandong/6/96	A/Hongkong/308/2014	A/Anhui-Lujiang/39/2018	A/Fujian-si ming/19/2021	A/Anhui-Langya/1144/2022
HA	T163N	T (35), N (13)	T	T	T	T	T	T	T	N	N
	H191N	N (48)	N	H	N	N	N	N	N	N	N
	T197D	T (17), D (31)	T	T	T	T	T	T	D	D	D
	T198V/A	T (28), V (15), A (5)	V	E	T	A	A	T	T	V	V
	Q234L	L (48)	Q	L	L	Q	Q	L	L	L	L
	Q235M	M (48)	Q	Q	Q	Q	Q	M	M	M	M
	D280G	D (36), G(12)	D	D	D	D	D	D	D	D	D
	N285S	S (48)	N	N	N	N	N	S	S	S	S
	R381K	K (48)	K	K	R	R	K	K	K	K	K
NA	Stalk 62 ~ 64	Del (48)	TEI	TEI	Del	Del	Del	Del	Del	Del	Del
	N70S	S (46), N (2)	S	N	S	S	S	S	S	N	S
	D369S/G	G (42), S (6)	D	D	D	D	D	S	S	S	S
	D401G/V	D (39), G(2), V(7)	D	D	D	D	D	D	D	D	E
	N402D	D (36), N (12),	N	N	N	N	N	D	N	N	N
	Q432H	Q (44), H (4)	Q	Q	Q	Q	Q	Q	Q	Q	Q
PB1	I368V	V (48)	I	I	I	I	I	V	V	V	V
	D622G	G (48)	G	G	G	G	G	G	G	G	G
PB2	V147I	V (22), I (26)	V	M	D	I	V	I	I	I	I
	I292V	I (6), V (42)	I	V	I	I	I	V	V	V	V
	A588V	A (5), V (43)	A	A	A	A	A	A	V	V	V
	E627V	E (27), V (21)	E	E	E	E	E	E	V	E	V
PA	K356R	R (48)	K	K	K	K	K	R	R	R	R
	S409N	N (48)	S	S	S	N	S	S	S	N	N
M1	N30D	D (48)	D	D	D	D	D	D	D	D	D
M2	S31N	S (2), N (46)	S	S	S	N	S	N	N	N	N
NP	I353V	I (4), V (44)	I	I	I	V	I	V	V	V	V
NS1	P42S	S (48)	S	S	S	S	S	S	S	S	S

Data in parentheses indicate the number of isolates harboring the mutation.

For the internal proteins, all isolates harbored the I368V and D622G mutations in PB1; exhibited V147I, I292V, A588V, and E627V mutations in PB2; carried the K356R and S409N mutations in PA; possesses the N30D mutation in M1 and the S31N mutation in M2; displayed the I353V mutation in NP; and harbored the P42S mutation in NS1, as shown in Table 3.

### 3.4 Potential glycosylation site analysis of H9N2 subtype isolate

In this study, the HA protein of the isolates harbored a total of eight potential N-linked glycosylation sites (at positions 29, 82, 141, 298, 305, 313, 492, and 551), six of which were consistently present (at positions 29, 82, 141, 298, 313, and 492). Compared with classical strains, all isolates lost the glycosylation site at position 218 (NRTF) and acquired an additional site at position 313 (NCSK). The glycosylation site pattern was largely consistent with that of human-derived strains, except that six isolates lost the site at position 551 (NGSC), and one isolate exhibited an alteration at the 305NISK glycosylation site, as shown in Table 4.

**Table 4:** Analysis of glycosylation sites in the HA protein of H9N2 subtype avian influenza virus isolates from external environments in western Zhejiang, 2014–2025.

Strain	29	82	141	218	298	305	313	492	551
Classical strains									
A/chicken/Beijing/1/94	NSTE	NPSC	NVTY	NRTF	NTTL	NVSK	–	NGTY	NGSC
A/quail/HongKong/G1/97	NSTE	NPSC	NVTY	NRTF	NSTL	NISK	–	NGTY	NGSC
A/duck/HongKong/Y439/97	NSTE	NPSC	NVTY	NRTF	NTTL	NVSK	–	NGTY	NGSC
A/duck/Hongkong/Y280/97	NSTE	NPSC	NVSY	NRTF	NTTL	NVSK	–	NGTY	-
Vaccine strains									
A/Chicken/Guangdong/SS/94	NSTE	NPSC	NVSY	NRTF	NTTL	NVSK	–	NGTY	NGSC
A/chicken/Shanghai/F/98	NSTE	NPSC	NVSY	NRTF	NTTL	NVSK	–	NGTY	NGSC
A/chicken/Shandong/6/96	NSTE	NPSC	NVSY	–	NTTL	NVSK	–	NGTY	NGSC
A/Hong Kong/308/2014	NSTE	NPSC	NVSY	–	NTTL	NVSK	NCSK	NGTY	NGSC
A/Anhui-Lujiang/39/2018	NSTE	NPSC	NVSY	–	NTTL	NVSK	NCSK	NGTY	NGSC
Human-derived strains									
A/Fujian-siming/19/2021	NSTE	NPSC	NVSY	–	NTTL	NVSK	NCSK	NGTY	NGSC
A/Anhui-Langya/1144/2022	NSTE	NPSC	NVSY	–	NTTL	NVSK	NCSK	NGTY	NGSC
48 isolates	NSTE (48)	NPSC (48)	NVSY (48)	–	NTTL (48)	NVSK (47), NISK (1)	NCSK (48)	NGTY (48)	NGSC (42)

– indicates absence of the glycosylation site. Data in parentheses indicate the number of isolates harboring the glycosylation site.

The NA protein of the isolates contained a total of nine potential N-linked glycosylation sites (at positions 44, 69, 86, 146, 200, 234, 264, 368, and 402), five of which were consistently present (at positions 69, 146, 200, 234, and 368). Some isolates lost glycosylation sites at positions 44 (7 isolates), 86 (1 isolate), and 402 (36 isolates), while three isolates acquired an additional site at position 264NISP. Notably, six isolates exhibited a glycosylation site pattern identical to that of the human-derived strain A/Anhui-Langya/1144/2022, all showing an alteration at the 368NSSR glycosylation site.

## 4 Discussion

Western Zhejiang is located on the southwestern flank of the Yangtze River Delta, The region features complex terrain and dense water networks, serving as an important stopover site and flyway for migratory birds. Intensive poultry farming, backyard poultry raising, and live poultry trade activities are widespread. These characteristics provide typical ecological conditions for the natural circulation and cross-species transmission of AIV, making the region a key area for avian influenza prevention and control in Zhejiang Province and even eastern China. This study revealed that the overall AIV positivity rate in the external environment of western Zhejiang was significantly higher than the average levels reported for Zhejiang Province and China as a whole (15, 16), suggesting that this region is a high-risk area for environmental enrichment of AIV. The co-circulation of multiple subtypes and mixed infections further increases the public health risk. In terms of temporal distribution, the positivity rates varied significantly across years, which may be attributed to multiple factors, including the coverage of avian influenza vaccination, migratory bird dynamics, and environmental and climatic changes (17). A distinct seasonal pattern was observed, with a peak in winter and spring, which is consistent with the high-incidence season for HPAIV (18). During winter and spring, low temperatures prolong the survival of the virus in the external environment, This period also coincides with the migratory bird season, when large numbers of waterfowl along the East Asian-Australasian Flyway congregate in the coastal wetlands of southeastern China (19), thereby increasing the risk of viral introduction and transmission. These findings also highlighted winter and spring as critical seasons for avian influenza surveillance and control. Trend analysis revealed a declining trend in the positivity rate of avian influenza viruses in the external environment of western Zhejiang after the COVID-19 pandemic. This decline is likely attributable to the synergistic effects of multiple factors, including the ancillary inhibitory effect of non-pharmaceutical interventions, the strengthening of live poultry market management regulations in Zhejiang Province, the immune barrier established through high-density compulsory vaccination, and natural fluctuations in viral ecology (20–22). Since 2022, Zhejiang Province has continuously implemented a compulsory avian influenza immunization program, maintaining a herd immunity density of over 90% year-round, a vaccination rate of 100% for all susceptible animals, and an antibody qualification rate consistently above 80% (22).

In terms of regional distribution, Kaihua County exhibited a significantly higher positivity rate than other areas. Which may be attributed to the county’s location at the borders of Zhejiang, Anhui, and Jiangxi provinces, its abundant wetland resources, and the dense concentration of stopover sites for migratory birds, suggesting that Kaihua County should be considered a key area for avian influenza prevention and control. Regarding the distribution by sampling site, the highest positivity rate was observed in urban and rural live poultry markets. This finding is closely associated with cross-infection due to the mixed trading of poultry from different species and origins in live poultry markets. These markets serve as critical nodes for the transmission of AIV in external environments and represent important sites for human exposure to AIV (23). During periods of high avian influenza activity, the timely closure of live poultry markets should be considered to reduce the risk of human infection. Swab samples collected from the surface of chopping boards used for slaughtering or displaying poultry showed a significantly higher positivity rate compared with other specimen types, which is consistent with previous studies (24). This finding highlighted the importance of timely cleaning and disinfection of chopping board surfaces to prevent cross-contamination. Multivariate analysis also indicated that that live poultry markets and slaughterhouses had the highest AIV contamination risk (*aOR* > 4.5), identifying them as key transmission nodes requiring targeted biosecurity. Significant district-level heterogeneity existed, with Kaihua County exhibiting nearly four-fold higher odds. Seasonality was pronounced (second quarter/third quarter reductions of 67 and 34% vs. first quarter), confirming a spring peak and supporting post-winter intervention timing. Collectively, environmental surveillance plays a critical role in monitoring viral circulation dynamics and identifying high-risk nodes prior to avian influenza outbreaks. Therefore, it is recommended that environmental surveillance be incorporated into the regional One Health routine surveillance network, establishing a prevention and control mechanism involving regular monitoring, risk assessment, and strategy optimization, thereby achieving a shift from “passive response” to “active early warning” (25).

In terms of subtype distribution, the H9 subtype was the predominant subtype in western Zhejiang, and the widespread occurrence of multiple mixed infections may increase the risk of viral reassortment. Although the H9N2 subtype is classified as LPAIV, its role as a donor of internal genes is becoming increasingly prominent. Previous studies have shown that 12 key strains of the H9N2 subtype, serving as internal gene donors, promote cross-species transmission of AIV by reducing the species barrier. Reducing the prevalence of H9N2 therefore represents an effective strategies to lower the risk of cross-species transmission of AIV (26). Timely elucidation of the molecular evolutionary characteristics of the H9N2 subtype AIV is essential for precise prevention and control of avian influenza and for ensuring public health security.

Phylogenetic analysis revealed that all H9N2 subtype isolates in this study belonged to the G57 genotype, consistent with the reassortment pattern of the dominant H9N2 genotype circulating in China since 2013. This genotype is characterized by its internal genes serving as donors that frequently reassort with other AIV subtypes, thereby facilitating the emergence of novel subtypes. Studies have shown that the internal genes of HPAIV such as H5N1, H7N9, and H10N8 originated from the G57 genotype (27–29), posing a significant threat to public health security. Among the internal genes, the PB2 and M genes belonged to the G1-like branch, while the PB1, PA, NP, and NS genes belonged to the F/98-like branch, further supporting the characterization of H9N2 subtype as a multi-source reassortant virus. The isolates in this study showed high homology with avian- or human-derived strains from eastern Chinese provinces including Zhejiang, Fujian, Jiangxi, and Anhui, confirming cross-provincial transmission of the H9N2 subtype in eastern China. Notably, the frequent poultry movements in the Yangtze River Delta region suggested the existence of a regional epidemic circulation, indicating wide-area viral gene exchange and creating favorable conditions for viral reassortment. Additionally, some isolates were closely related to the human-derived strains A/Anhui-Langya/1144/2022 and A/Fujian-siming/19/2021, clustering within the same evolutionary clade, suggesting a potential risk of cross-species transmission that warrants close attention.

Studies have demonstrated that key amino acid mutations in the H9N2 subtype AIV play important roles in viral pathogenicity and host specificity (30, 31). Therefore, In-depth analysis of its mutation patterns is critical for evaluating the zoonotic potential and public health risk of the virus. In this study, multiple key amino acid mutations were identified in the HA, NA, and internal proteins of the isolates, Notably, some of which were consistent with those found in human-derived strains, suggesting a potential risk of cross-species transmission. Specifically, Mutations at the receptor-binding sites (RBS) in the HA protein, including H191N, Q234L, and Q235M, have been reported to enhance the binding affinity of the virus to human-type receptors (*α*-2,6-linked sialic acid receptors) (32, 33). Additionally, Mutations at antigenic epitopes D280G and N285S not only reduce vaccine-induced immune protection and promote immune escape but also synergize with RBS mutations to enhance binding to α-2,6-linked sialic acid receptors (34, 35). A deletion at positions 62–64 in the stalk region of the NA protein was present in all isolates; this deletion represents an important molecular marker of viral adaptation from aquatic to terrestrial poultry and is associated with increased transmission efficiency (36). Mutations at the hemadsorption site, including D369S/G, D401G/V, and N402D, may affect viral release efficiency (37, 38). Regarding drug resistance-associated sites, only the N70S mutation was identified, which may reduce susceptibility to zanamivir (9). In contrast, the S31N mutation in the M2 protein, associated with amantadine resistance, was prevalent in most isolates, suggesting potential amantadine resistance among H9N2 subtype viruses in western Zhejiang (39). Mutations in the PB2 protein, including I292V and E627V, have been demonstrated to enhance polymerase activity in mammalian cells and are key molecular markers of adaptation to mammalian hosts (40, 41). Additional Mutations such as PB1-I368V and PA-K356R/S409N further augment viral polymerase activity and replication efficiency (42, 43). The NS1-P42S mutation enhances the ability of the virus to inhibit the host interferon system, thereby increasing viral replication and pathogenicity in mammalian hosts (44). Collectively, these adaptive mutations constitute the molecular basis for H9N2 subtype viruses to cross the species barrier, indicating that H9N2 viruses in western Zhejiang are continuously evolving toward mammalian adaptation and pose a potential risk of cross-species transmission. Furthermore, specific alterations in glycosylation sites of the HA and NA proteins were observed in some isolates, which were highly consistent with those of human-derived strains, further confirming the convergence of molecular characteristics between H9N2 isolates in western Zhejiang and human-derived strains.

This study has some additional limitations. First, there is variability in the methodologies used for external environmental surveillance of avian influenza viruses, and no internationally recognized standardized protocol currently exists. Sampling is often concentrated in poultry-dense locations (e.g., live poultry markets, intensive farms, migratory bird habitats), making uniform random sampling difficult and potentially introducing systematic bias. Second, low viral loads and high sample contamination levels in environmental specimens challenge virus detection, meaning the true level of contamination may be underestimated. Third, due to the long study period and broad geographic coverage, complete vaccination data were unavailable, precluding assessment of vaccine impacts on viral evolution and transmission. Future studies should integrate multi-sectoral data, including vaccination records, into surveillance networks for more comprehensive risk assessment. Fourth, the potential cross-species transmission risk of H9N2 viruses is inferred solely from genetic sequence analysis without functional validation. Recognition of these limitations will guide optimization of surveillance strategies, support scientific interpretation of data, and enhance the efficiency and credibility of the monitoring system.

## 5 Conclusion

This study systematically characterizes the epidemiological and genetic evolutionary dimensions of AIV in the external environment of western Zhejiang from 2014 to 2025. Regarding epidemiological characteristics, the external environment exhibited a high overall AIV positivity rate (34.64%), pronounced spatiotemporal clustering (winter–spring peak and marked district-level heterogeneity), and the concurrent co-circulation of multiple subtypes including H5, H7, and H9, with the H9 subtype predominating throughout the study period. These findings confirm the high-risk status of this region and identify live poultry markets, slaughterhouses, and chopping board surfaces as critical transmission nodes. Regarding genetic and evolutionary characteristics, all 48 H9N2 isolates belonged to the G57 genotype and had accumulated multiple mammalian-adaptive mutations, including receptor-binding site alterations (T163N, H191N, T197D, T198V, Q234L, Q235M), NA stalk deletion (62–64 aa), and internal protein substitutions (PB1-I368V, D622G. PB2-E627V. M2-S31N), indicating a potential risk of cross-species transmission. Cross-provincial genetic linkages were also confirmed, with the H9N2 isolates showing high sequence identity to strains from Zhejiang, Fujian, Jiangxi, Anhui, Shanghai, and Guangdong, consistent with regional epidemic circulation driven by inter-provincial poultry trade in the Yangtze River Delta. From a One Health perspective, These findings underscore the necessity of integrating environmental, poultry, and human surveillance data into a unified early-warning framework. Future efforts should focus on developing an integrated animal-environment-human surveillance system based on molecular markers to provide a scientific basis for early warning and precise prevention and control of human infections with avian influenza.

## Data Availability

The datasets presented in this article are not readily available because Zhejiang Influenza Surveillance Information System are encrypted and closed to public access. The datasets used and/or analyzed during the current study are available from the corresponding author upon reasonable request. Requests to access these datasets should be directed to Ruijun Yang, qzyangruijun@126.com.
